# Axl Deficiency Promotes the Neuroinvasion of Japanese Encephalitis Virus by Enhancing IL-1α Production from Pyroptotic Macrophages

**DOI:** 10.1128/JVI.00602-20

**Published:** 2020-08-17

**Authors:** Zhao-Yang Wang, Zi-Da Zhen, Dong-Ying Fan, Cheng-Feng Qin, Dai-Shu Han, Hong-Ning Zhou, Pei-Gang Wang, Jing An

**Affiliations:** a Department of Microbiology, School of Basic Medical Sciences, Capital Medical University, Beijing, China; b Department of Virology, State Key Laboratory of Pathogen and Biosecurity, Beijing Institute of Microbiology and Epidemiology, Beijing, China; c Institute of Basic Medical Sciences, Chinese Academy of Medical Sciences, Peking Union Medical College, Beijing, China; d Yunnan Provincial Center of Arbovirus Research, Yunnan Provincial Key Laboratory of Vector-borne Diseases Control and Research, Yunnan Institute of Parasitic Diseases, Pu'er, Yunnan, China; e Center of Epilepsy, Beijing Institute for Brain Disorders, Beijing, China; University of Kentucky College of Medicine

**Keywords:** Axl, Japanese encephalitis virus, interleukin-1α, macrophages, pyroptosis

## Abstract

Japanese encephalitis virus (JEV) is a flavivirus that causes Japanese encephalitis (JE), which has an unclear pathogenesis. Despite vaccination, thousands of deaths attributed to JE are reported annually. In this study, we report that mice deficient for Axl, a receptor tyrosine kinase that plays multiple roles in flaviviral infection, displayed greater mortality upon JEV infection. The effect of Axl deficiency on JEV infection was mediated by markedly elevated serum interleukin-1α (IL-1α) levels, which devastated the blood-brain-barrier and promoted viral neuroinvasion within 24 h postinfection. Using an *in situ* infection model, we showed that dead macrophages were the primary source of observed increased serum IL-1α levels. Axl deficiency enhanced cell death and caused pyroptosis in 80% of JEV-infected macrophages by disrupting phosphatidylinositol 3-kinase (PI3K)-Akt signaling. Intriguingly, the primary effector released by pyroptotic macrophages in our model was IL-1α rather than IL-1β. Finally, we assessed the effect of an IL-1α antagonist and demonstrated that it effectively prevented the incidence of JE. Our results indicate that Axl plays a protective role in JEV infection, identify IL-1α released by pyroptotic macrophages as a crucial factor promoting JEV neuroinvasion, and suggest that an IL-1α antagonist may be a candidate for JE therapy.

**IMPORTANCE** Japanese encephalitis virus (JEV) is a mosquito-borne flavivirus that causes Japanese encephalitis (JE), the most commonly diagnosed viral encephalitis worldwide. The fatality rate of JE is 20%, and nearly half of the surviving patients develop neuropsychiatric sequelae. Axl is a receptor tyrosine kinase that plays multiple roles in flaviviral infections. Currently, the involvement of Axl in JEV infection remains enigmatic. In this study, we demonstrate that Axl impedes the pathogenesis of severe JE in mice by maintaining blood-brain-barrier (BBB) integrity and restricting viral neuroinvasion. Furthermore, serum IL-1α is a key mediator of this process and is primarily released by JEV-infected pyroptotic macrophages to elicit BBB breakdown, while an IL-1α antagonist can effectively reduce the incidence of severe JE. Our work uncovers the protective role of Axl in antagonizing severe JE and shows that the use of an IL-1α antagonist may be a promising tactic to prevent severe JE.

## Abstract (precis)

Japanese encephalitis virus (JEV) is a mosquito-borne flavivirus that causes Japanese encephalitis (JE), the most commonly diagnosed viral encephalitis worldwide. The fatality rate of JE is 20%, and nearly half of the surviving patients develop neuropsychiatric sequelae. Axl is a receptor tyrosine kinase that plays multiple roles in flaviviral infections. Currently, the involvement of Axl in JEV infection remains enigmatic. In this study, we demonstrate that Axl impedes the pathogenesis of severe JE in mice by maintaining blood-brain-barrier (BBB) integrity and restricting viral neuroinvasion. Furthermore, serum IL-1α is a key mediator of this process and is primarily released by JEV-infected pyroptotic macrophages to elicit BBB breakdown, while an IL-1α antagonist can effectively reduce the incidence of severe JE. Our work uncovers the protective role of Axl in antagonizing severe JE and shows that the use of an IL-1α antagonist may be a promising tactic to prevent severe JE.

## INTRODUCTION

Japanese encephalitis virus (JEV), a member of the same genus (*Flavivirus*) as dengue virus (DENV), Zika virus (ZIKV), and West Nile virus (WNV), is transmitted by *Culex* mosquitos and causes Japanese encephalitis (JE), which is endemic in 24 countries and is the most important viral encephalitis worldwide (1). Although vaccination has dramatically reduced the incidence of JE, approximately 67,900 cases of JE still occur annually (2). Unfortunately, even after surviving the encephalitis, nearly half of patients bear permanent neuropsychiatric sequelae (1). Moreover, due to a number of unfavorable factors, such as insufficient vaccination coverage and global warming, the incidence of JE is still increasing in some developing countries (3). Nevertheless, to date, we have neither enough knowledge regarding the mechanism of JEV infection nor a specific therapy for JE.

As a member of the receptor tyrosine kinase family Tyro3, Axl, and Mertk (TAM), which recognizes phosphatidylserines (PtdSers) exposed on the surface of apoptotic cells and enveloped viruses (4, 5), Axl has been proposed to be an entry receptor for various enveloped viruses, such as Lassa virus (6), vaccinia virus (7), and ebolavirus (8). Regarding flaviviruses, a group to which JEV belongs, Axl has been thought to serve as an entry receptor for ZIKV, DENV, and WNV (9–12). However, accumulating evidence has shown that its roles in flaviviral infection are diverse and vary based on the virus and host type. For example, Axl mediates DENV entry via Gas6 bridging (13), while Axl knockout has no impact on WNV replication in target organs but increases its neuroinvasion (14). The involvement of Axl in ZIKV infection is more complicated and controversial. Although Axl promotes ZIKV infection in many cell types *in vitro*, its knockout has no impact on ZIKV replication or pathogenesis in mouse models, human neural progenitor cells, or cerebral organoids (15–17). Recently, Axl knockout mice were shown to be resistant to ZIKV pathogenesis in an age-dependent manner, corresponding to lower prointerleukin-1β (pro-IL-1β) production and decreased apoptosis in microglia (18). The complexity of the contributions of Axl to flaviviral infection is potentially associated with its versatile functions. Axl not only mediates the removal of dead cells by phagocytes and maintains immune and inflammatory homeostasis but also regulates all forms of programed cell death (19–21). Therefore, Axl has additional roles beyond that of a receptor in flaviviral infection, and its roles in infection need to be investigated on an individual basis for each flavivirus.

In this study, using Axl-deficient mice, we discovered that Axl suppresses JE pathogenesis by inhibiting IL-1α production from pyroptotic macrophages induced by JEV. IL-1α mediates the early neuroinvasion of JEV by disrupting blood-brain-barrier (BBB) integrity, and an IL-1α antagonist was shown to potently reduce the incidence of severe JE. The results of our study revealed the crucial roles of Axl and IL-1α in JEV infection and suggest that IL-1α antagonists may be candidate drugs for JE therapy.

## RESULTS

### Axl deficiency promotes higher mortality after JEV infection.

**FIG 1:**
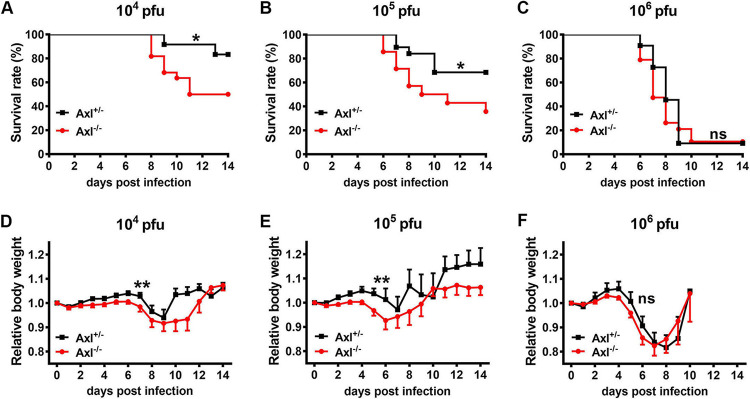
Effects of Axl on Japanese encephalitis virus (JEV) infection. Four-week-old Axl^−/−^ and Axl^+/−^ mice received an intraperitoneal (i.p.) injection of different doses of JEV (10^4^, 10^5^, and 10^6^ PFU). (A to C) Survival curves of Axl^+/−^ and Axl^−/−^ mice infected with 10^4^ PFU (A) (*n* = 12 for Axl^+/−^, *n* = 22 for Axl^−/−^), 10^5^ PFU (B) (*n* = 19 for Axl^+/−^, *n* = 14 for Axl^−/−^), and 10^6^ PFU of JEV (C) (*n* = 11 for Axl^+/−^, *n* = 19 for Axl^−/−^). The survival curves were compared by log-rank test. (D to F) Body weight changes of Axl^+/−^ and Axl^−/−^ mice infected with 10^4^ PFU (D) (*n* = 26 for Axl^+/−^, *n* = 19 for Axl^−/−^), 10^5^ PFU (E) (*n* = 7 for Axl^+/−^, *n* = 9 for Axl^−/−^), and 10^6^ PFU of JEV (F) (*n* = 11 for Axl^+/−^, *n* = 19 for Axl^−/−^). The body weight changes were compared by two-way ANOVA. Data are expressed as means ± SEM; *, *P* < 0.05; **, *P* < 0.01; ns, no significance (*P* > 0.05); each result is the representative of three independent experiments.

To study the role of Axl in JEV infection, 4-week-old Axl-deficient mice (Axl^−/−^) and their littermate control mice (Axl^+/−^) received an intraperitoneal (i.p.) injection of different doses of JEV (10^4^, 10^5^, and 10^6^ PFU per mouse). Axl^+/−^ mouse littermates were used as controls because they are more comparable with respect to genetic background, age, and nutrition status, all of which have important impacts on mortality upon JEV infection according to our experience. At 6 to 8 days postinfection (dpi), 17% to 91% of mice, which depended on the dose, began to lose weight and became sluggish. As the disease progressed, these mice gradually manifested ruffled fur, hunched back, and limb paralysis and finally died from encephalitis. At a dose of 10^4^ PFU of JEV, the mortality of the Axl^−/−^ mice was 50% (11/22), which was significantly higher than the 17% (2/12) observed in the Axl^+/−^ mice (*P* < 0.05) (Fig. 1A), demonstrating that the Axl^−/−^ mice were more susceptible to JEV infection. As the infection dose increased, the mortality gap between the Axl^−/−^ and Axl^+/−^ mice decreased and vanished when 10^6^ PFU of JEV was injected, which led to death in more than 90% of mice (Fig. 1B and C). Compared to the Axl^+/−^ mice, the Axl^−/−^ mice displayed faster and more severe body weight loss after disease onset at all doses of JEV (*P* < 0.01 for 10^4^ and 10^5^ PFU, *P* = 0.06 for 10^6^ PFU) (Fig. 1D to F). These results indicated that Axl may be a protective factor against JE pathogenesis that improves the survival of mice after JEV infection.

**FIG 2:**
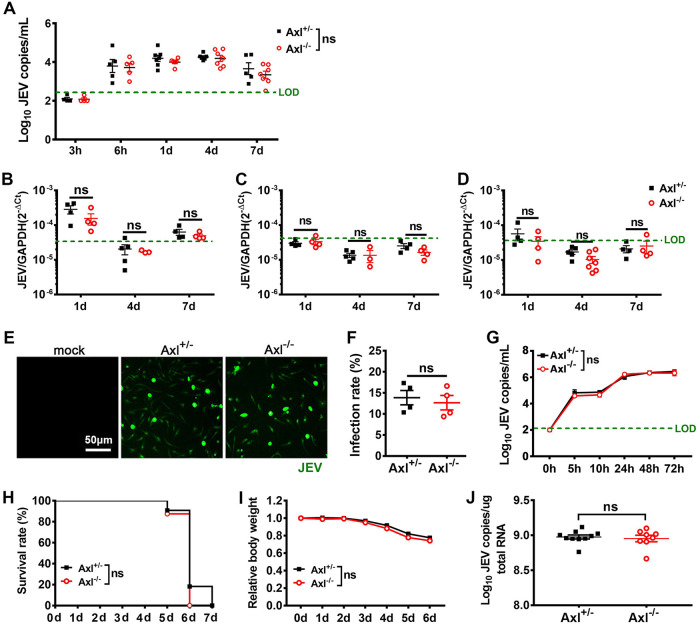
Effects of Axl on peripheral and brain JEV infection. (A to D) Four-week-old Axl^−/−^ and Axl^+/−^ mice received an i.p. injection of 10^4^ PFU of JEV. Viral loads in serum (A), spleen (B), liver (C), and kidney (D) at the indicated time points postinfection. LOD (green dashed line), limit of detection. Each dot denotes a mouse, and the viral loads were compared by two-way ANOVA and multiple *t* tests. (E to G) Primary peritoneal macrophages were infected with JEV at an MOI of 1. (E) Immunofluorescent (IF) staining of JEV antigens (green) in peritoneal macrophages at 24 hpi. (F) Infection rates of peritoneal macrophages at 24 hpi. *n* = 4 for each group, and the infection rates were compared by unpaired *t* test. (G) Viral loads in supernatants of peritoneal macrophages. *n* = 4 for each group, and the data were compared by two-way ANOVA and multiple *t* tests. (H to J) Four-week-old Axl^−/−^ mice (*n* = 8) and Axl^+/−^ mice (*n* = 12) were intracerebrally injected with 10^3^ PFU of JEV. (H) Survival curves were compared by log-rank test. (I) Body weight changes were compared by two-way ANOVA. (J) Viral loads in brain at 6 dpi, each dot denotes a mouse, and the viral loads were compared by unpaired *t* test. All the data are expressed as the means ± SEM; ns, no significance (*P* > 0.05); each result is the representative of three independent experiments.

One possible explanation for these results is that Axl inhibited JEV replication. To test this hypothesis, we measured viral RNA loads in serum and peripheral organs and analyzed JEV infection in primary macrophages, which are reported to be the primary target cells during JEV infection. After i.p. infection with JEV, viral RNA was detectable in serum at 6 h postinfection (hpi) and lasted for at least 7 days (Fig. 2A). The Axl^−/−^ and control mice demonstrated similar levels of viral RNA in serum (Fig. 2A) and all assayed peripheral organs at different time points (Fig. 2B to D). Peritoneal macrophages isolated from the Axl^−/−^ and control mice were infected with an equivalent dose of JEV *in vitro* but showed no difference regarding viral infection rate and replication dynamics (Fig. 2E to G). To further test if Axl is only a restrictive factor in the brain, we injected JEV directly into mouse brains. All mice died within 7 days (Fig. 2H), which was accompanied by similar body weight loss (Fig. 2I) and viral RNA loads in the brain (Fig. 2J). Thus, Axl is not likely to be a restrictive factor that directly inhibits JEV replication. Considering that the Axl^−/−^ mice had similar viral loads in peripheral organs but were at a greater risk of developing encephalitis, Axl possibly plays a protective role by regulating the BBB permeability for JEV.

### Axl deficiency increases the probability of JEV brain infection.

**FIG 3:**
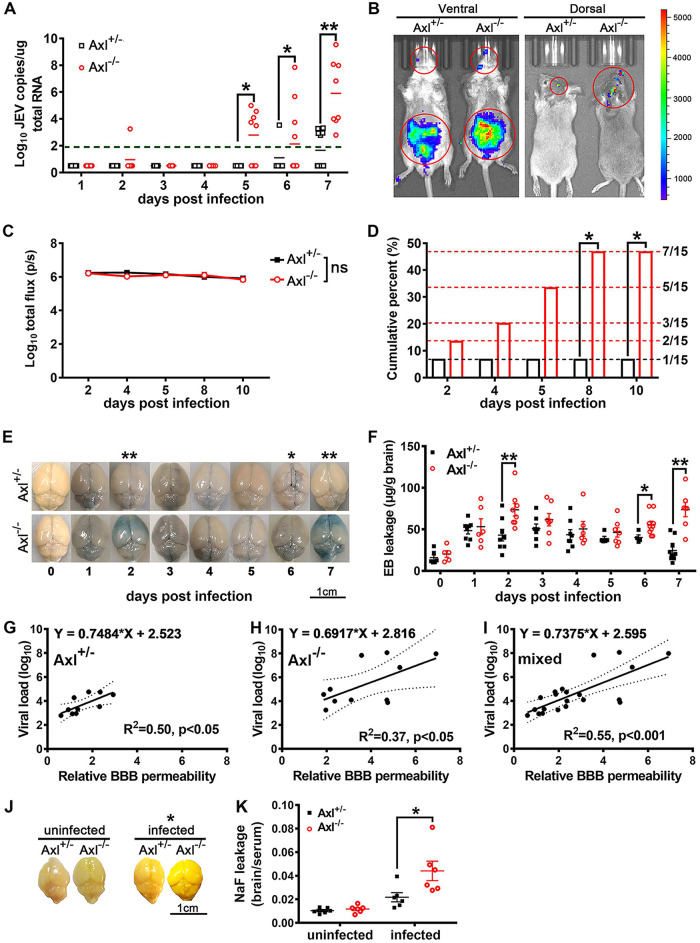
Effects of Axl on JEV neuroinvasion and blood-brain barrier (BBB) permeability. Four-week-old Axl^−/−^ and Axl^+/−^ mice received an i.p. injection of 10^4^ PFU of JEV (A and E to K) or 5 × 10^4^ PFU of Rluc-JEV for bioluminescent imaging (BLI) (B to D). (A) Shows viral loads in brains; each dot denotes a mouse, and viral loads were compared by two-way ANOVA and multiple *t* tests. (B) Representative BLI image of Rluc-JEV infection. (C) Quantification of the BLI signal intensity in peritoneal cavity; *n* = 15 for each group, and the data were compared by two-way ANOVA and multiple *t* tests. (D) Cumulative percentage of the mice with Rluc-JEV entry into brain; *n* = 15 for each group, and the data were compared by Fisher’s exact test. (E) Representative image of Evans blue (EB) leakage into brain parenchyma. (F) Quantification of EB leakage into brain parenchyma after JEV infection; *n* = 5 to 8 for each group, and the data were compared by two-way ANOVA and multiple *t* tests. (G to I) Correlation analysis of BBB permeability and brain viral load in Axl^+/−^ mice (G), Axl^−/−^ mice (H), and mixed mice (I); each dot denotes a mouse, and the data were analyzed by linear regression model. (J) Representative image of sodium fluorescein (NaF) leakage into brain parenchyma. (K) Quantification of NaF leakage into brain parenchyma under uninfected and JEV-infected (7 dpi) conditions; each dot denotes a mouse, and the data were compared by multiple *t* tests. All the data are expressed as the means ± SEM; *, *P* < 0.05; **, *P* < 0.01; ns, no significance (*P* > 0.05); each result is the representative of three independent experiments.

To investigate if Axl protects brains from JEV infection, we measured the brain viral loads of the Axl^−/−^ and control mice from 1 to 7 dpi after i.p. infection with JEV. Viral RNA was barely detectable in mouse brains until 5 dpi (Fig. 3A). The Axl^−/−^ mice displayed a higher positive rate of JEV and greater viral loads in the brain than control mice (Fig. 3A), indicating that Axl^−/−^ mouse brains were more accessible to JEV. We further observed the target cell usage of JEV in mouse brains (cerebral cortex) through immunofluorescence (IF) staining of NeuN (a specific marker for neurons), GFAP (a specific marker for astrocytes), Iba1 (a specific marker for microglia), and JEV antigens, but no differences between the Axl^−/−^ and control mice were observed (data not shown), suggesting the protective role of Axl is achieved by inhibiting JEV entry into the brain rather than altering its cell tropism in the brain.

To confirm the central nervous system (CNS) entry-inhibition role of Axl, mice received an i.p. injection of 5 × 10^4^ PFU of Renilla luciferase-harboring JEV (Rluc-JEV), and the luciferase substrate was injected at different time points to allow viral replication and spread to be continuously monitored via bioluminescent imaging (BLI). Rluc-JEV was first detected in the abdomens (Fig. 3B), the site of infection, in all mice by 2 dpi and later in the brains of a few mice (Fig. 3B). The Axl^−/−^ and control mice exhibited no difference in signal intensity in the abdomens (Fig. 3C). However, more Axl^−/−^ mice showed luciferase-positive brains (7/15) than the control mice (1/15) by 10 dpi (Fig. 3D), in agreement with the results obtained with the wild-type JEV. Taken together, the results show that Axl deficiency remarkably increased the probability of JEV entry into the brain.

### Axl deficiency increases BBB permeability at an early stage of JEV infection.

To understand the mechanism underlying the protective role of Axl against JE pathogenesis, we utilized an Evans blue (EB) leakage test to gauge the permeability of the BBB after JEV infection, which is the key structure preventing viral entry into the brain. In the uninfected groups, both the Axl^−/−^ and control mice showed very little and comparable EB leakage into the brain parenchyma, indicating that Axl deficiency has no effect on BBB permeability under physiological conditions (Fig. 3E and F, 0 dpi). After JEV infection, EB leakage into the brain parenchyma significantly increased and reached the first peak at 2 dpi, when the Axl^−/−^ mice showed significantly enhanced EB leakage compared with that observed in control mice, indicating that Axl deficiency promoted the BBB permeability at the early phase of infection (Fig. 3E and F, 2 dpi). After 2 dpi, both the Axl^−/−^ and control mice showed gradually decreased EB leakage into the brain parenchyma until 6 dpi, when EB leakage in the Axl^−/−^ mice increased again, while the control mice showed a continual decrease in EB leakage (Fig. 3E and F, 2 to 7 dpi). The Axl^−/−^ mice showed significantly enhanced EB leakage compared with the control mice at 6 and 7 dpi, indicating that Axl deficiency also promoted BBB permeability at the late phase of infection (Fig. 3E and F, 6 and 7 dpi). Moreover, in both the Axl^−/−^ and Axl^+/−^ mice, there was a strong positive linear correlation between BBB permeability and brain viral load (slope = 0.6917, R^2^ = 0.37, *P* < 0.05 for Axl^−/−^ mice; slope = 0.7484, R^2^ = 0.50, *P* < 0.05 for Axl^+/−^ mice; and slope = 0.7375, R^2^ = 0.55, *P* < 0.001 for all mice) (Fig. 3G to I), suggesting that greater BBB permeability facilitates JEV entry into brains. We also used a fluorescein leakage test to confirm the results obtained in the EB leakage test. In the uninfected groups, all mice showed slight but comparable fluorescein leakage into the brain parenchyma. In contrast, the Axl^−/−^ mice showed significantly greater fluorescein leakage than the Axl^+/−^ mice after JEV infection (7 dpi) (Fig. 3J and K). These data indicate that Axl deficiency significantly increases BBB permeability and consequently promotes JEV entry into brain.

**FIG 4:**
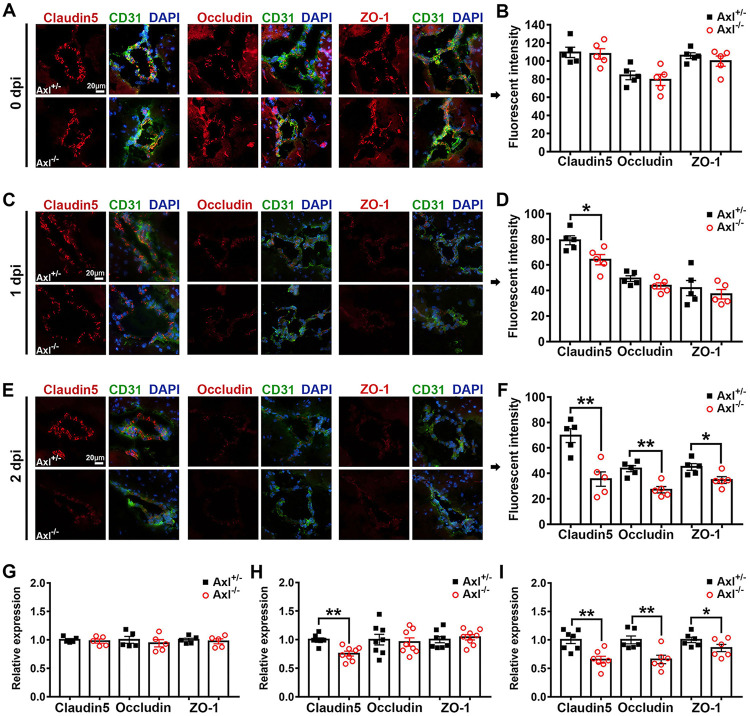
Effects of Axl on the integrity of tight junctions in the brain. Four-week-old Axl^−/−^ and Axl^+/−^ mice received an i.p. injection of 10^4^ PFU of JEV. (A, C, and E) IF staining of claudin-5, occludin, and ZO-1 (tight junction proteins, red); CD31 (microvascular endothelial cell marker, green); and 4′,6-diamidino-2-phenylindole (DAPI; nucleus, blue) in brain at 0 dpi (A), 1 dpi (C), and 2 dpi (E), where each image is the representative from 3 to 5 mice; scale bar = 20 μm. (B, D, and F) Quantification of the fluorescent intensity of claudin-5, occludin, and ZO-1 in brain at 0 dpi (B), 1 dpi (D), and 2 dpi (F); each dot denotes a mouse, and the data were compared by unpaired *t* test. (G to I) Relative mRNA abundance of claudin-5, occludin, and ZO-1 in mouse brains at 0 dpi (G), 1 dpi (H), and 2 dpi (I), normalized to the average of Axl^+/−^ mice; each dot denotes a mouse, and the data were compared by unpaired *t* test. All the data are expressed as the means ± SEM; *, *P* < 0.05; **, *P* < 0.01; each result is the representative of three independent experiments.

In the results described above, we noted that the Axl^−/−^ mice displayed greater BBB permeability at an early stage of JEV infection, which is much earlier than the appearance of encephalitis. To confirm this phenomenon and investigate the underlying mechanisms, we elucidated the dynamic changes in the expression and distribution of three important tight junction (TJ) proteins in the brain, namely, claudin-5, occludin, and ZO-1, using IF staining. In the uninfected mice (0 dpi), claudin-5 displayed the strongest signal and was located at the interfaces between adjacent endothelial cells, as revealed using an antibody against CD31 (a specific marker of vascular endothelial cells) (Fig. 4A). Occludin and ZO-1 displayed a similar distribution as claudin-5 (Fig. 4A). No difference between the Axl^−/−^ and control mice was observed with respect to the IF staining intensity of all three TJ proteins (Fig. 4B). After JEV infection, in both the Axl^−/−^ and control mice, the IF staining intensity of the three TJ proteins was apparently reduced (Fig. 4C). Remarkably, the Axl^−/−^ mice showed a lower IF staining intensity of claudin-5 than the control mice at 1 dpi (Fig. 4D). At 2 dpi, the Axl^−/−^ mice displayed an even lower IF staining intensity of claudin-5, occludin, and ZO-1 than the control mice (Fig. 4E and F). We also detected the levels of claudin-5, occludin, and ZO-1 mRNA in the brains of the Axl^−/−^ and control mice at 0, 1, and 2 dpi using quantitative real-time PCR (qRT-PCR), and we observed that the RNA abundance of the three TJ proteins was consistent with their protein levels detected by IF staining (Fig. 4G to I). Taken together, our results suggest that prior to the occurrence of encephalitis, the expression of TJ proteins in the brains of Axl^−/−^ mice have already been disrupted, making it easier for JEV to penetrate the BBB.

### IL-1α mediates early JEV neuroinvasion in Axl-deficient mice.

**FIG 5:**
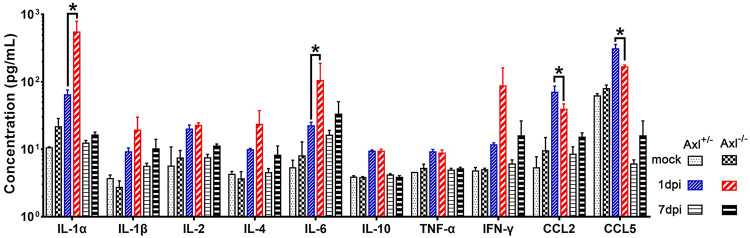
Cytokine profiling in serum. Four-week-old Axl^−/−^ and Axl^+/−^ mice received an i.p. injection of 10^4^ PFU of JEV or 200 μl of PBS (mock) per mouse, and serum samples were obtained at 1 and 7 dpi. A panel of cytokines, including IL-1α, IL-1β, IL-2, IL-4, IL-6, IL-10, TNF-α, IFN-γ, CCL2, and CCL5 in sera were detected by a bead-based immunoassay (Aimplex); *n* = 2 for mock, and *n* = 6 for 1 and 7 dpi; and the data were compared by two-way ANOVA and multiple *t* tests. All the data are expressed as means ± SEM; *, *P* < 0.05; this result is the representative of three independent experiments.

As changes in BBB permeability and TJ protein levels rapidly occurred after JEV infection, we wondered whether Axl^−/−^ mice triggered the release of some humoral factors to disrupt BBB integrity soon after JEV infection. To address the question, we measured the serum levels of a panel of cytokines (IL-1α, IL-1β, IL-2, IL-4, IL-6, IL-10, tumor necrosis factor alpha [TNF-α], interferon gamma [IFN-γ], CCL2, and CCL5) that may affect BBB permeability at 1 and 7 dpi. Without JEV infection, no obvious difference was observed in the serum levels for any of the assayed cytokines between the Axl^−/−^ and Axl^+/−^ mice (Fig. 5, mock). In contrast, upon JEV infection, the serum levels of all measured cytokines increased at 1 dpi and returned to a lower level at 7 dpi that was comparable to those observed in the mock-treated group (Fig. 5, mock, 1 and 7 dpi). Compared with the Axl^+/−^ mice, the Axl^−/−^ mice showed higher levels of IL-1α, IL-1β, IL-2, IL-4, IL-6, and IFN-γ but lower levels of CCL2 and CCL5 in serum at 1 dpi (Fig. 5, 1 dpi). Of the six cytokines with increased levels, IL-1α (1 dpi) and IL-6 (1 dpi) were greatly increased in Axl^−/−^ mice (Fig. 5, 1 dpi), suggesting that they may be the mediators responsible for the observed BBB breakdown.

**FIG 6:**
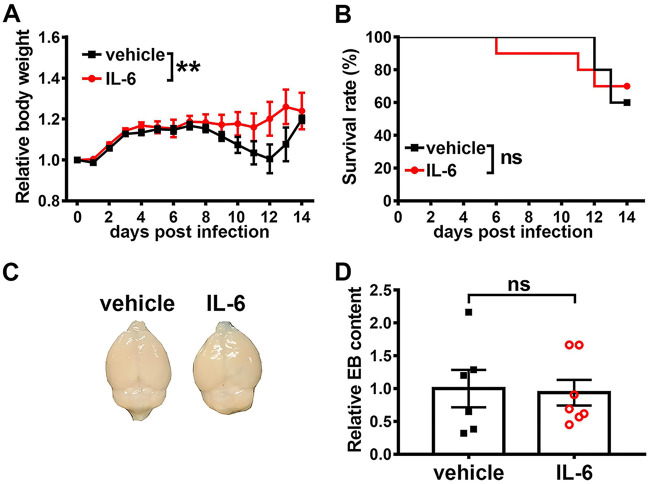
Effects of IL-6 on JEV infection outcome and BBB permeability. (A and B) Four-week-old Axl^−/−^ mice received an i.p. injection of recombinant mouse IL-6 (60 ng per mouse, *n* = 10) or vehicle (sterile normal saline, *n* = 10) 30 min prior to an i.p. injection of 10^4^ PFU of JEV, and then they were treated daily with IL-6 (60 ng per mouse) or vehicle (sterile normal saline). (A) Body weight changes were compared by two-way ANOVA. (B) Survival curves were compared by log-rank test. (C and D) Four-week-old Axl^−/−^ mice received a single i.p. injection of IL-6 (60 ng per mouse) or vehicle (sterile normal saline), and BBB permeability was measured 6 h later by an EB leakage test. (C) Representative image showing EB leakage into brain parenchyma. (D) Quantification of EB content in brain, normalized to the average of vehicle-treated mice, where each dot denotes a mouse, and the data were compared by unpaired *t* test. All the data are expressed as means ± SEM; **, *P* < 0.01; ns, no significance (*P* > 0.05); each result is the representative of three independent experiments.

To further investigate the effect of IL-1α and IL-6 on JE pathogenesis, we treated JEV-infected Axl^−/−^ mice with IL-1α, IL-6, or vehicle (sterile normal saline) daily. Compared with the vehicle-treated mice, the IL-6-treated mice showed slightly higher body weights (Fig. 6A) but no differences in mortality (Fig. 6B) or BBB permeability (Fig. 6C and D), which excluded it as a crucial contributor to JE pathogenesis in Axl^−/−^ mice.

**FIG 7:**
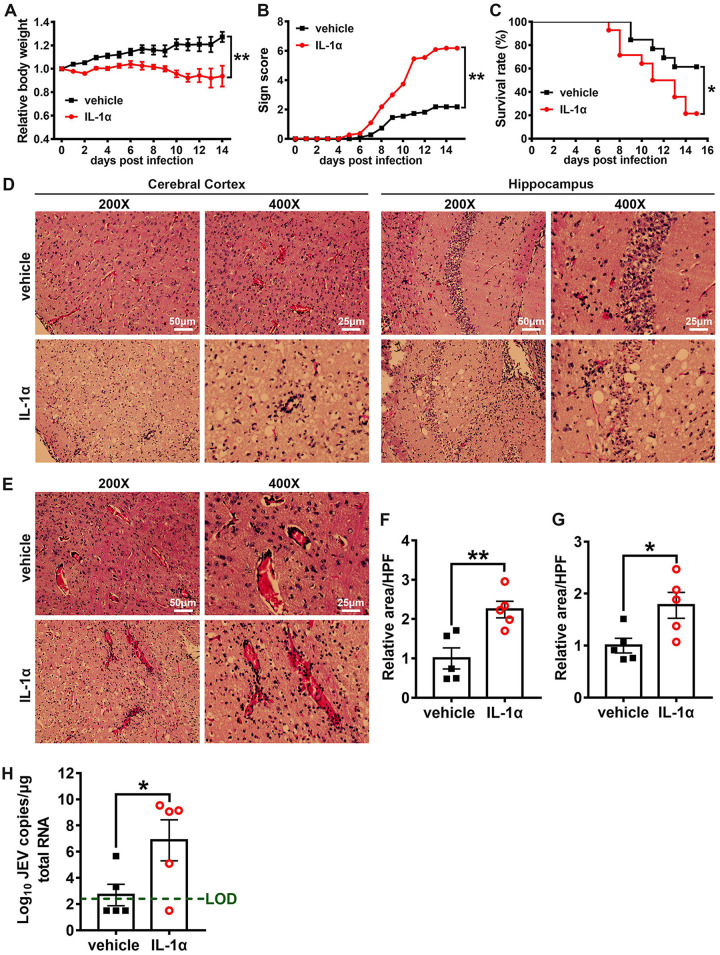
Effects of IL-1α on JEV infection outcome. Four-week-old Axl^−/−^ mice received an i.p. injection of recombinant mouse IL-1α (200 ng per mouse, *n* = 14) or vehicle (sterile normal saline, *n* = 13) 30 min prior to an i.p. injection of 10^4^ PFU of JEV and then were treated daily with IL-1α or vehicle. (A) Body weight changes were compared by two-way ANOVA. (B) Scores of the infection signs, which were compared by rank-sum test. (C) Survival curves were compared by log-rank test. (D) Pathological lesions in cerebral cortex and hippocampus at 7 dpi. (E) Perivascular infiltration of inflammatory cells in cerebral cortex at 7 dpi. (F and G) Quantification of the area of neuron death (F) and the area of inflammatory cell infiltration (G) in cerebral cortex per high power filed (HPF), normalized to the average of vehicle-treated mice; *n* = 5 for each group, and the data were compared by unpaired *t* test. (H) Viral load in mouse brains at 7 dpi; green dashed line denotes the limit of detection, *n* = 5 for each group, and the data were compared by unpaired *t* test. All the data are expressed as the means ± SEM; *, *P* < 0.05; **, *P* < 0.01; each result is the representative of three independent experiments.

In contrast, mice treated with IL-1α showed more severe body weight loss and JE signs than vehicle-treated mice (Fig. 7A and B), and the mortality rate in the IL-1α-treated group (11/14) was significantly greater than that observed for the vehicle-treated group (5/13) (Fig. 7C). In microscopic observations, JEV infection induced obvious neuroinflammation, including neuron cell death, angiectasis, perivascular cuffing, and inflammatory cell infiltration (Fig. 7D and E). Moreover, IL-1α-treated mice showed significantly larger areas of neuron death (Fig. 7D and F) and inflammatory cell infiltration than the vehicle-treated mice (Fig. 7E and G) at 7 dpi, suggesting that IL-1α treatment enhanced JEV-induced brain lesions. We also detected the viral load in mouse brains by qRT-PCR and observed that the IL-1α-treated mice showed greater viral loads in the brain than the vehicle-treated mice (Fig. 7H). Taken together, these results show that IL-1α promotes the pathogenesis of severe JE by promoting the invasion of JEV into the brain.

**FIG 8:**
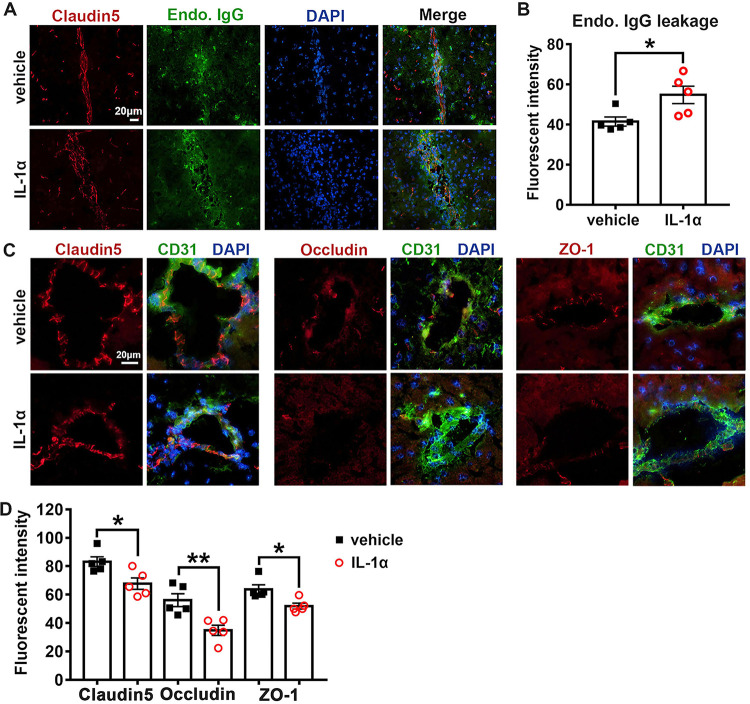
Effects of IL-1α on BBB permeability and tight junction integrity during JEV infection. (A) Representative image displaying endogenous IgG (green) leakage into brain parenchyma. Claudin-5 (red) was used to position BBB; DAPI was used to show nucleus; scale bar = 20 μm. (B) Quantification of endogenous IgG leakage into brain parenchyma at 7 dpi; *n* = 5, and the data were compared by unpaired *t* test. (C) Representative image depicting brain claudin-5, occludin, and ZO-1 (red); CD31 (microvascular endothelial cell marker, green); and DAPI (nucleus, blue); scale bar = 20 μm. (D) Quantification of the staining intensity of claudin-5, occludin, and ZO-1 in the cerebral cortex at 7 dpi; *n* = 5, and the data were compared by unpaired *t* test. All the data are expressed as the means ± SEM; *, *P* < 0.05; **, *P* < 0.01; each result is the representative of three independent experiments.

To elucidate the impact of IL-1α on BBB permeability in JEV-infected mice, we evaluated BBB permeability by quantifying endogenous IgG leakage and assessing the expression and distribution of claudin-5, occludin, and ZO-1 in mouse brains by IF staining. In contrast to the vehicle-treated mice, the IL-1α-treated mice showed significantly increased endogenous IgG leakage into the brain parenchyma (Fig. 8A and B). In agreement with this result, the expression of claudin-5, occludin, and ZO-1 in the IL-1α-treated mice was conspicuously lower than that observed in the vehicle-treated mice, and the distribution of the three TJ proteins was more severely disrupted in the IL-1α-treated mice than in the vehicle-treated mice (Fig. 8C and D). Taken together, these results indicate that IL-1α but not IL-6 disrupted the BBB integrity and promoted JEV invasion into brains.

**FIG 9:**
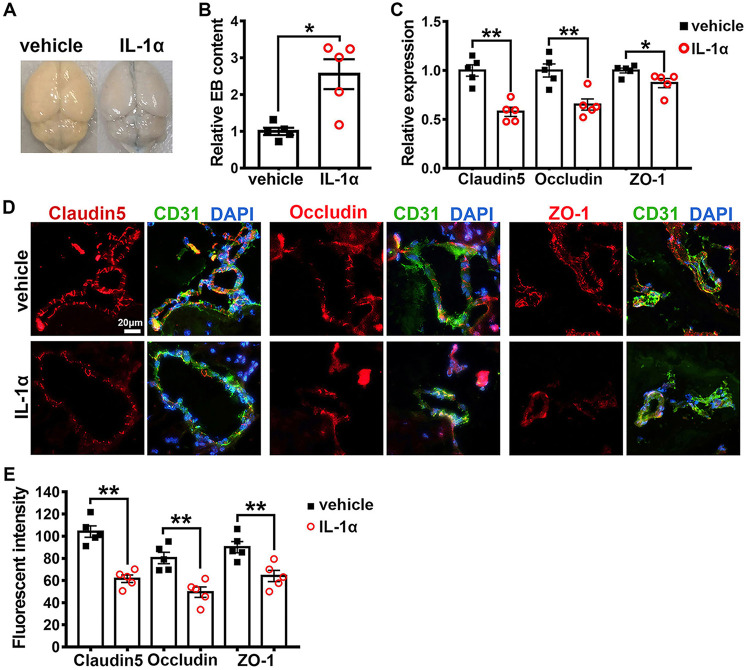
Effects of IL-1α alone on BBB permeability and tight junction integrity. Four-week-old Axl^−/−^ mice received an i.p. injection of recombinant mouse IL-1α (200 ng per mouse) or vehicle (sterile normal saline). At 6 h posttreatment, BBB permeability was gauged by an EB leakage test, and brain tight junction proteins were observed by IF staining. (A) Representative image showing EB leakage into brain parenchyma. (B) BBB permeability change after IL-1α treatment; *n* = 5, and the data were compared by unpaired *t* test. (C) mRNA abundance of claudin-5, occludin, and ZO-1 in mouse brains after IL-1α treatment, normalized to the average of vehicle-treated mice; *n* = 5, and the data were compared by unpaired *t* test. (D) IF staining of claudin-5, occludin, and ZO-1 (red); CD31 (microvascular endothelial cell marker, green); and DAPI (nucleus, blue); scale bar = 20 μm. (E) Quantification of the IF staining intensity of claudin-5, occludin, and ZO-1; *n* = 5 for each group, and the data were compared by unpaired *t* test. All the data are expressed as the means ± SEM; *, *P* < 0.05; **, *P* < 0.01; each result is the representative of three independent experiments.

To ensure IL-1α was the factor inducing BBB and TJ breakdown at the early stage of JEV infection, an i.p. injection of a mouse recombinant IL-1α was administered to Axl^−/−^ mice, and its direct effect on BBB permeability and brain TJs was assessed at 6 h posttreatment, when viral RNA was first detected in serum. BBB permeability was measured by EB leakage assays and by quantifying the levels of claudin-5, occludin, and ZO-1 mRNA by qRT-PCR and assessing their distribution by IF staining. Compared with the vehicle-treated mice, the IL-1α-treated mice showed greater EB leakage into the brain parenchyma (2.6-fold), indicating that IL-1α alone could immediately increase BBB permeability (Fig. 9A and B). Furthermore, the IL-1α-treated mice showed significantly lower mRNA levels of claudin-5, occludin, and ZO-1 (Fig. 9C) and much weaker IF staining intensity for these TJ proteins than was observed in the vehicle-treated mice (Fig. 9D and E). Moreover, unlike in the vehicle-treated mice, where the TJ proteins clearly located at the interface between two neighboring endothelial cells, the TJ proteins in the IL-1α-treated mice were dispersed and lost their typical distribution patterns (Fig. 9D), indicating that IL-1α alone could rapidly disrupt the distribution of TJ proteins and increase the BBB permeability.

### IL-1α is released by dead JEV-infected *in situ* peritoneal macrophages.

**FIG 10:**
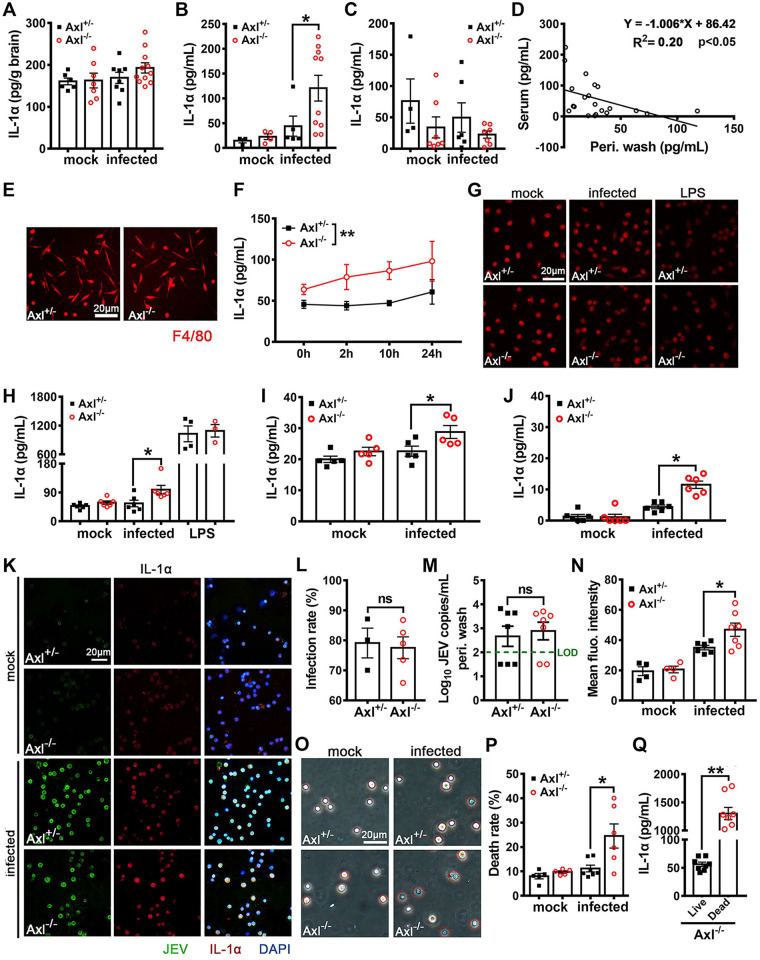
Effect of Axl on IL-1α production during JEV infection. (A to D) Four-week-old Axl^−/−^ and Axl^+/−^ mice received an i.p. injection of 10^4^ PFU of JEV. IL-1α content in the brain (A), serum (B), and peritoneal wash (C) at 24 hpi; each dot denotes a mouse, and the data were compared by unpaired *t* test. (D) Correlation analysis of IL-1α content in serum and peritoneal wash at 24 hpi; the data were analyzed by linear regression model. (E to H) Peritoneal macrophages were isolated from Axl^−/−^ and Axl^+/−^ mice, cultured, and infected with JEV (MOI = 1) or stimulated with LPS (1 μg/ml) *in vitro*. (E) IF staining of F4/80 (marker of macrophages) in untreated Axl^−/−^ and Axl^+/−^ peritoneal macrophages. Scale bar = 20 μm. (F) Dynamics of IL-1α contents in the supernatants of peritoneal macrophages; *n* = 4 for each group, and the data were compared by two-way ANOVA. (G) IF staining of intracellular IL-1α in peritoneal macrophages at 24 hpi. Scale bar = 20 μm. (H) IL-1α contents in the supernatants of peritoneal macrophages at 24 hpi; *n* = 3 to 6 for each group, and the data were compared by unpaired *t* test. (I and J) Murine cerebral microvascular endothelial cells (bEND.3) and primary splenocytes were infected with JEV (MOI, 1). (I) IL-1α contents in the supernatants of bEND.3 cells at 24 hpi; *n* = 5, and the data were compared by unpaired *t* test. (J) IL-1α contents in the supernatants of primary splenocytes at 24 hpi; *n* = 6, and the data were compared by unpaired *t* test. (K) IF staining of JEV antigens and IL-1α inside the *in situ* peritoneal macrophages at 24 hpi. Scale bar = 20 μm. (L) Infection rates of the *in situ* peritoneal macrophages at 24 hpi; *n* = 3 for Axl^+/−^, and *n* = 5 for Axl^−/−^; the data were compared by unpaired *t* test. (M) Viral loads in peritoneal washes at 24 hpi; *n* = 7 for each group, and the data were compared by unpaired *t* test. (N) IF staining intensity of intracellular IL-1α in the *in situ* peritoneal macrophages at 24 hpi; *n* = 4 to 7 for each group, and the data were compared by unpaired *t* test. (O) Trypan blue staining of the *in situ* peritoneal macrophages at 24 hpi. Scale bar = 20 μm. (P) Cell death rates of the *in situ* peritoneal macrophages at 24 hpi; *n* = 5 to 7, and the data were compared by unpaired *t* test. (Q) IL-1α content in the supernatant of live and hypotonically dead Axl^−/−^ peritoneal macrophages; *n* = 8 for each group, and the data were compared by unpaired *t* test. All the data are expressed as the means ± SEM; *, *P* < 0.05; **, *P* < 0.01; ns, no significance (*P* > 0.05); each result is the representative of three independent experiments.

To identify the source of IL-1α in the JEV-infected mice, we measured the IL-1α content in the brain, serum, and peritoneal wash at 24 hpi. No significant alteration in the brain IL-1α content was observed (Fig. 10A). Consistent with previous measurements, serum IL-1α levels sharply increased (Fig. 10B). Remarkably, peritoneal wash levels of IL-1α showed a decreasing tendency after JEV infection (Fig. 10C), and there was a negative correlation with respect to the IL-1α content between the serum and peritoneal wash (Fig. 10D). The above data suggested that serum IL-1α was primarily derived from the peritoneal cavity rather than the brain, and it was probably achieved by releasing the preexisting IL-1α inside cells rather than increasing IL-1α expression. The serum IL-1α content in Axl^−/−^ mice was much greater than that observed in the control mice (Fig. 10B), whereas the IL-1α content in the peritoneal wash samples from Axl^−/−^ mice was lower than that detected in the control mice (Fig. 10C), suggesting that Axl deficiency accelerated the release of IL-1α by peritoneal cells.

To elucidate the mechanism underlying the Axl-mediated regulation of IL-1α release by peritoneal cells, we isolated mouse peritoneal cells and infected them with JEV *in vitro*. Most of the peritoneal cells were F4/80 positive (Fig. 10E), suggesting they were macrophages. In the uninfected groups, Axl^−/−^ and control macrophages showed similar IL-1α content in the supernatant. However, the IL-1α content in the supernatant of Axl^−/−^ macrophages immediately increased after infection, which was maintained at higher levels than that of the control macrophages at all tested time points, suggesting that Axl deficiency promotes the release of IL-1α (Fig. 10F). However, the magnitude of the increase of IL-1α in the supernatant of Axl^−/−^ macrophages was not enough to explain the drastic rise in serum IL-1α levels. Moreover, at 24 hpi, the expression of IL-1α inside peritoneal macrophages did not decrease significantly (Fig. 10G), and the IL-1α content in the supernatant was slightly elevated (Fig. 10H). We also measured IL-1α production by mouse brain microvascular endothelial cells (bEND.3) and primary splenocytes *in vitro* in the absence of Axl signaling, which was achieved by Axl deficiency or inhibition with R428, but these cells produced even less IL-1α than the peritoneal macrophages (Fig. 10I and J). Compared with the JEV-infected cells, lipopolysaccharide (LPS)-stimulated peritoneal macrophages showed a conspicuous decrease in intracellular IL-1α expression (Fig. 10G) and a sharp increase in the IL-1α content in the supernatant (Fig. 10H). IL-1α can be released by either LPS induction or cell death (22, 23). Since IL-1α release by peritoneal macrophages after JEV infection occurred through a different mechanism than LPS stimulation, it was speculated that it may have been released by dead cells as an alarm molecule.

We noticed that the peritoneal macrophages cultured *in vitro* were not highly susceptible to JEV infection (Fig. 2E and F), and no detectable death was observed after infection. Thereafter, we avoided the *in vitro* infection model and instead directly isolated the peritoneal macrophages from JEV-infected mice and made smears, which closely reflected the *in situ* infection characteristics of peritoneal macrophages. We observed that *in situ* macrophages were highly susceptible to JEV infection, with an infection rate of up to 80% (Fig. 10K and L). There was no significant difference in the infection rate between the Axl^−/−^ and control macrophages (Fig. 10L), which was in accordance with the similar viral titers observed in the peritoneal wash samples (Fig. 10M) and with the previous results (Fig. 2F and G). In both the Axl^−/−^ and control macrophages, IL-1α was largely localized to the nucleus, which was consistent with previous reports (24). At 24 hpi, the expression of intracellular IL-1α significantly increased, and the Axl^−/−^ macrophages showed slightly greater expression of IL-1α than that observed in the control macrophages (Fig. 10K and N). These results suggest that IL-1α is released not only by induction but also by immediately liberating preexisting intracellular IL-1α.

To assess whether serum IL-1α was released by dead macrophages after JEV infection, we first used the Trypan blue (TB) staining method to discriminate live cells (unstained, TB^−^) and dead cells (blue stained, TB^+^) (Fig. 10O), At 24 hpi, the proportion of TB^+^ cells in the peritoneal wash samples of the Axl^−/−^ mice was significantly increased, much more than that observed for the control mice (Fig. 10O and P). To determine whether additional IL-1α can be released after cell death, we used a hypotonic treatment to induce the death of Axl^−/−^ peritoneal macrophages, observing that IL-1α levels in the supernatant increased by more than 2 orders of magnitude (Fig. 10Q). Taken together, these results suggest that macrophages lacking Axl die more readily after JEV infection, which triggers a substantial release of IL-1α from cells.

### Axl deficiency promotes the pyroptosis of JEV-infected peritoneal macrophages.

**FIG 11:**
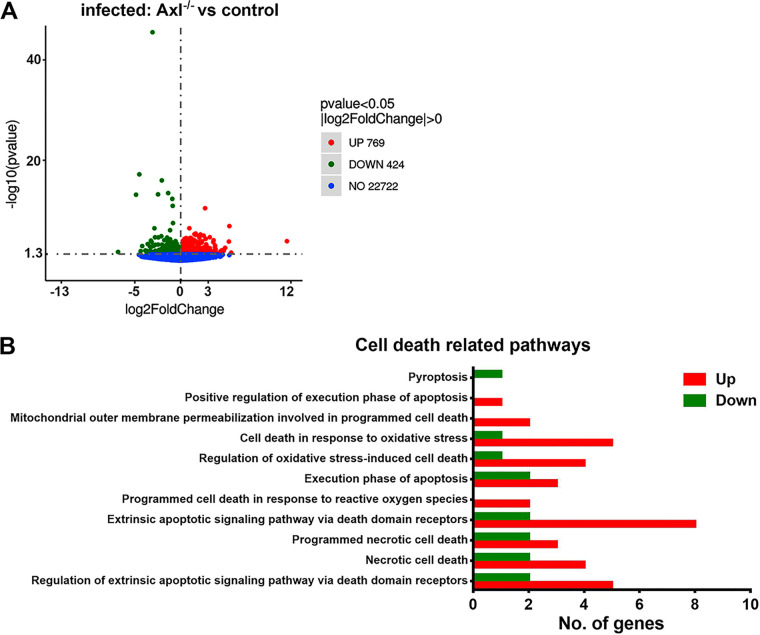
Transcriptomic analysis of the cell death pathways in the *in situ* peritoneal macrophages. Four-week-old Axl^−/−^ and Axl^+/−^ mice received an i.p. injection of 10^4^ PFU of JEV. At 24 hpi, the *in situ* peritoneal macrophages were isolated and subjected to transcriptomic sequencing. (A) Volcano plot showing the upregulated, downregulated, and unchanged genes. JEV-infected Axl^−/−^ macrophages versus JEV-infected Axl^+/−^ macrophages; each dot denotes a specific gene; *n* = 5 for each group. (B) Gene Ontology (GO) enrichment analysis of the cell death-related biological processes; JEV-infected Axl^−/−^ macrophages versus JEV-infected Axl^+/−^ macrophages; *n* = 5 for each group.

To further elucidate the mode of death of JEV-infected macrophages, we isolated peritoneal macrophages from JEV-infected mice at 24 hpi and analyzed transcriptomic changes through transcriptome sequencing (RNA-Seq) analysis. Hundreds of differentially expressed genes were identified between the Axl^−/−^ and control macrophages after infection (Fig. 11A). Many differentially expressed genes were enriched in cell death-related biological processes, such as necrosis, apoptosis, and pyroptosis (Fig. 11B). Remarkably, compared with the control macrophages, most of these cell death-related genes in the Axl^−/−^ macrophages were upregulated after infection, suggesting that Axl deficiency enhanced the transcription of cell death-related genes (Fig. 11B). We also assessed the transcription of IL-1α, IL-1β, and IL-1 receptor antagonist (IL-1Ra), observing that only IL-1β was upregulated in JEV-infected Axl^−/−^ macrophages (Table 1). After infection, the transcription of caspase-1 and fasdermin D (GSDMD) (key mediators of pyroptosis) was notably enhanced but showed no difference between the Axl^−/−^ and control macrophages (Table 1). Although the transcription of RIPK1 (a key mediator of necroptosis) was unchanged, its suppressor BIRC3 showed greater transcription in Axl^−/−^ macrophages than in control macrophages after infection (Table 1). The transcription of caspase-8, caspase-9, and caspase-3 (all are crucial regulators of apoptosis and pyroptosis) was enhanced after infection, but no differences were observed between the Axl^−/−^ and control macrophages (Table 1). The expression of the proapoptotic gene *BCL-10* was upregulated in the Axl^−/−^ macrophages after infection (Table 1). In addition, *Noxo1*, which promotes the generation of ROS and cell death, was more expressed in the Axl^−/−^ macrophages than that observed in the control macrophages after infection (Table 1). PI3K-Akt signaling is an important pathway that promotes cell survival. After infection, the transcription levels of PI3K and Akt were similar in the Axl^−/−^ and control macrophages (data not shown), but the PI3K inhibitors PI3KIP1 and WDR91 as well as the Akt inhibitor TRIB3 showed increased expression in the Axl^−/−^ macrophages compared with that observed in the control macrophages (Table 1), suggesting that during infection, PI3K-Akt signaling may be inhibited in Axl^−/−^ macrophages.

**TABLE 1:** Transcription levels of IL-1 signaling-related genes and cell death-related genes^*a*^

Gene	Transcription level by treatment group			
	Mock		JEV	
	Axl^+/−^	Axl^−/−^	Axl^+/−^	Axl^−/−^
IL-1α	1.00 ± 0.19	1.85 ± 0.48	3.63 ± 0.59	3.89 ± 0.77
IL-1β	1.00 ± 0.18	1.07 ± 0.55	6.91 ± 0.84	23.55 ± 10.02^*b*^
IL-1Ra	1.00 ± 0.10	1.04 ± 0.11	15.02 ± 1.63	16.92 ± 5.71
Caspase-1	1.00 ± 0.14	0.80 ± 0.04	2.33 ± 0.08	2.03 ± 0.19
GSDMD	1.00 ± 0.11	0.93 ± 0.06	2.11 ± 0.02	2.00 ± 0.13
RIPK1	1.00 ± 0.05	0.94 ± 0.05	1.05 ± 0.02	1.02 ± 0.06
BIRC3	1.00 ± 0.05	0.94 ± 0.08	0.69 ± 0.02	0.84 ± 0.03^*c*^
Caspase-8	1.00 ± 0.07	0.95 ± 0.05	2.19 ± 0.11	2.17 ± 0.11
Caspase-9	1.00 ± 0.01	0.96 ± 0.01	1.13 ± 0.04	1.10 ± 0.02
Caspase-3	1.00 ± 0.04	1.20 ± 0.08	2.37 ± 0.09	2.21 ± 0.20
BCL-10	1.00 ± 0.04	0.99 ± 0.06	0.95 ± 0.02	1.08 ± 0.03^*b*^
Noxo1	1.00 ± 0.06	1.17 ± 0.11	0.46 ± 0.05	0.80 ± 0.04^*c*^
PI3KIP1	1.00 ± 0.09	0.88 ± 0.12	0.48 ± 0.03	0.72 ± 0.12^*b*^
WDR91	1.00 ± 0.03	0.92 ± 0.02	0.87 ± 0.02	1.00 ± 0.03^*b*^
TRIB3	1.00 ± 0.34	0.41 ± 0.11	0.60 ± 0.05	1.15 ± 0.24^*b*^

a The transcription levels of specific genes were normalized to the average vFPKM (fragments per kilobases of transcript per million fragments mapped) of mock Axl^+/−^ macrophages and are expressed as mean ± SEM. Data between two groups were analyzed by unpaired *t* test (JEV, Axl^−/−^ versus Axl^+/−^). *n* = 5 for each group.

b *P* < 0.05.

c *P* < 0.01.

**FIG 12:**
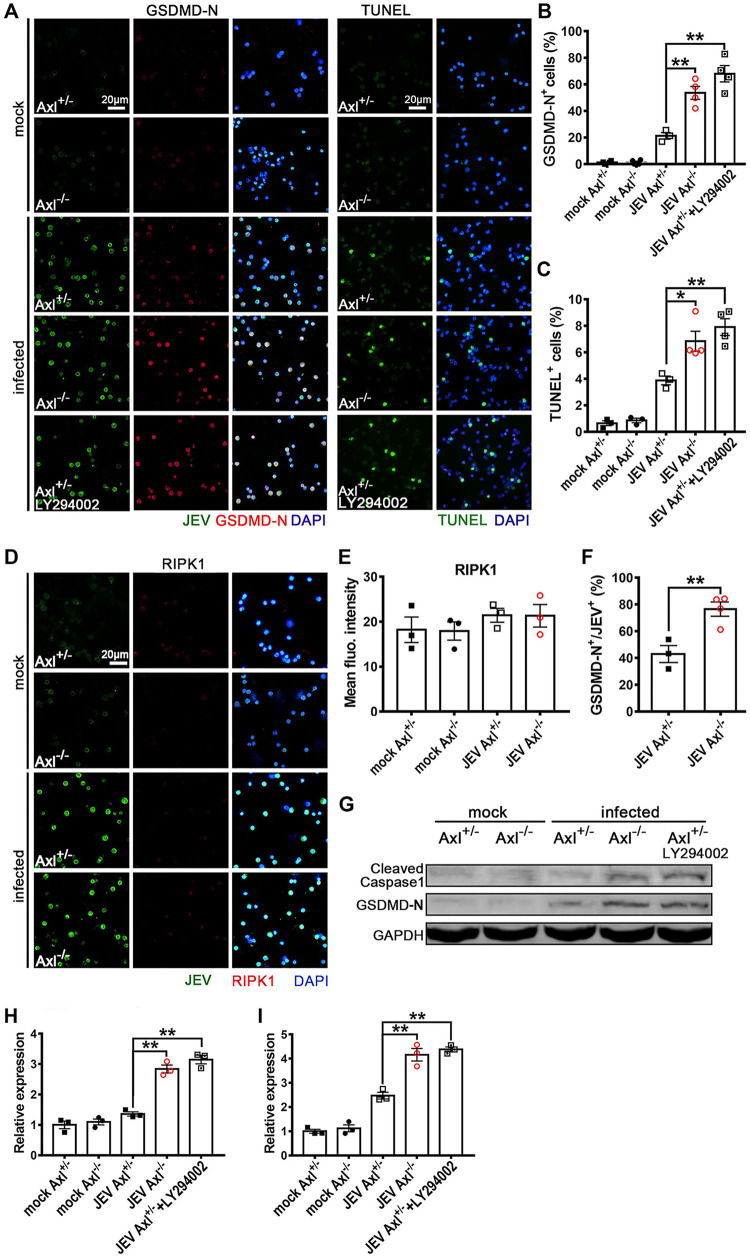
Effect of Axl on the cell death mode of *in situ* peritoneal macrophages. Four-week-old Axl^−/−^ and Axl^+/−^ mice received an i.p. injection of 10^4^ PFU of JEV. Specifically, for PI3K inhibition in the *in situ* Axl^+/−^ peritoneal macrophages, an i.p. injection of LY294002 (1.25 mg per mouse) was administered 1 h prior to i.p. infection. At 24 hpi, the *in situ* peritoneal macrophages were isolated. (A) IF staining of GSDMD-N (marker of pyroptosis) and TUNEL staining (marker of apoptosis) of the *in situ* peritoneal macrophages. (B) Pyroptosis (GSDMD-N^+^) rates of the *in situ* peritoneal macrophages; *n* = 3 to 4 for each group; the data were compared by unpaired *t* test. (C) Apoptosis (TUNEL^+^) rates of the *in situ* peritoneal macrophages; *n* = 3 to 4 for each group; the data were compared by unpaired *t* test. (D) IF staining of RIPK1 (marker of necroptosis) in the *in situ* peritoneal macrophages. (E) Quantification of the fluorescence intensity of RIPK1 in the *in situ* peritoneal macrophages; *n* = 3 for each group; the data were compared by unpaired *t* test. (F) Pyroptosis (GSDMD-N^+^) rate of JEV-infected (JEV^+^) *in situ* peritoneal macrophages; *n* = 3 to 4 for each group; the data were compared by unpaired *t* test. (G) Immunoblotting detection of cleaved caspase-1 and GSDMD-N in the *in situ* peritoneal macrophages at 24 hpi. GAPDH was used as a loading control; *n* = 3. (H and I) Quantification of the gray density of cleaved caspase-1 (H) and GSDMD-N (I), normalized to the average of mock-treated Axl^+/-^ macrophages; *n* = 3 for each group; the data were compared by unpaired *t* test. All the data are expressed as the means ± SEM; *, *P* < 0.05; **, *P* < 0.01; each result is the representative of three independent experiments.

**FIG 13:**
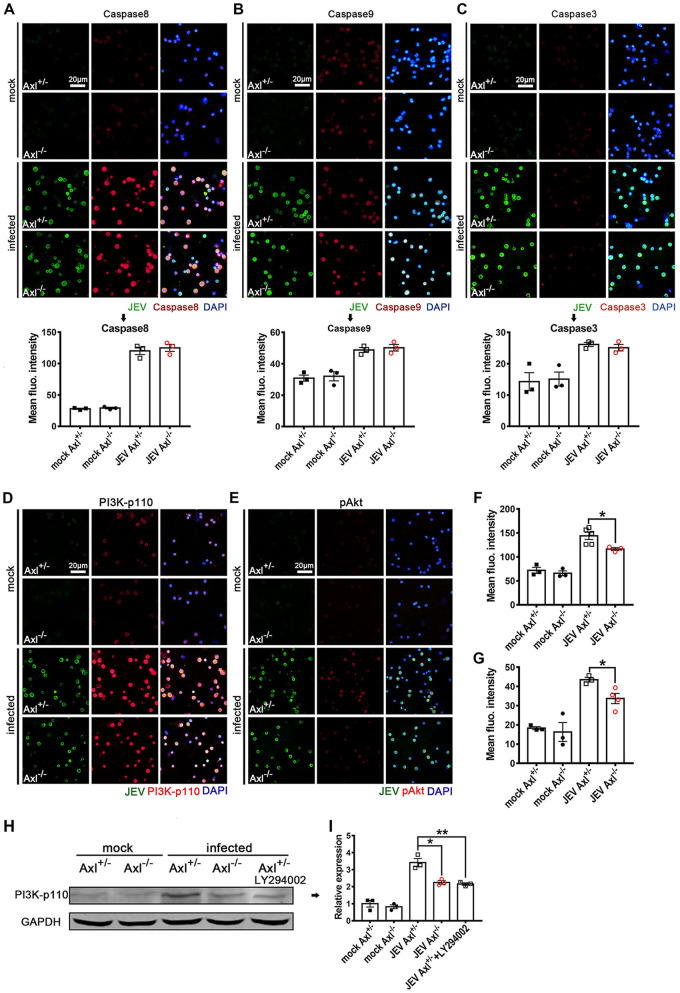
Effects of Axl on the expression of cell death regulatory proteins in the *in situ* peritoneal macrophages. Four-week-old Axl^−/−^ and Axl^+/−^ mice received an i.p. injection of 10^4^ PFU of JEV. At 24 hpi, the *in situ* peritoneal macrophages were isolated. (A to C) IF staining and quantification of caspase-8 (extrinsic apoptosis initiator) (A), caspase-9 (intrinsic apoptosis initiator) (B), and caspase-3 (apoptosis executor) (C), where these genes are also crucial regulators of pyroptosis; *n* = 3 for each group; the data were compared by unpaired *t* test. Scale bar = 20 μm. (D and E) IF staining of PI3K-p110 (D) and phosphorylated Akt (pAkt) (E) of the *in situ* peritoneal macrophages. Scale bar = 20 μm. (F and G) Quantification of the fluorescent intensity of PI3K-p110 (F) and pAkt (G) of the *in situ* peritoneal macrophages; *n* = 3 to 4 for each group; the data were compared by unpaired *t* test. (H) Immunoblotting detection of PI3K-p110 in the *in situ* peritoneal macrophages at 24 hpi. GAPDH is used as a loading control. (I) Quantification of the gray density of PI3K-p110, normalized to the average of mock-treated Axl^+/-^ macrophages; *n* = 3 for each group; the data were compared by unpaired *t* test. All the data are expressed as the means ± SEM; *, *P* < 0.05; **, *P* < 0.01; each result is the representative of three independent experiments.

Subsequently, we verified the RNA-Seq revealed cell death pathways (apoptosis, pyroptosis, and necroptosis) using IF staining. At 24 hpi, the proportion of GSDMD-N-terminal^+^ (a marker of pyroptosis) cells and terminal deoxynucleotidyltransferase-mediated dUTP-biotin nick end labeling (TUNEL)^+^ (a marker of apoptosis) cells increased significantly (Fig. 12A to C) but that of RIPK1^+^ (a marker of necroptosis) cells did not change significantly (Fig. 12D and E), indicating that the JEV-infected peritoneal macrophages primarily underwent pyroptosis and apoptosis rather than necroptosis, which was consistent with the RNA-Seq results. The pyroptosis and apoptosis rates in the Axl^−/−^ macrophages were much greater than those observed in the control mice (Fig. 12B and C), suggesting that Axl deficiency promotes the pyroptosis and apoptosis of JEV-infected macrophages. Remarkably, pyroptosis was the predominant death mode, with approximately 80% of JEV-infected Axl^−/−^ macrophages undergoing pyroptosis (Fig. 12F). We also assessed the expression of cleaved caspase-1 (another maker of pyroptosis) and GSDMD-N in the *in situ* peritoneal macrophages by immunoblotting and observed that, consistent with the IF staining results, Axl^−/−^ macrophages showed greater expression of the cleaved caspase-1 and GSDMD-N than the control macrophages after JEV infection (Fig. 12G to I). In subsequent experiments, caspase-8, a key molecule regulating cell death mode, was observed to be significantly upregulated in JEV-infected macrophages, but no significant difference in caspase-8 expression was observed between the Axl^−/−^ and control macrophages (Fig. 13A). The changes in caspase-9 and caspase-3 levels were similar to that observed for caspase-8 (Fig. 13B and C).

Axl has been shown to promote cell survival and inhibit cell apoptosis and pyroptosis by activating PI3K-Akt signaling (25, 26), and the RNA-Seq results also indicated that PI3K-Akt signaling was inhibited in Axl^−/−^ macrophages. To confirm this result, we examined the expression of PI3K-p110 and phosphorylated Akt (pAkt) in JEV-infected *in situ* macrophages using IF staining (Fig. 13D and E), observing that the expression of PI3K-p110 (Fig. 13D and F) and pAkt (Fig. 13E and G) were significantly lower in Axl^−/−^ macrophages than in control macrophages. Moreover, the PI3K inhibitor LY294002 could effectively reduce the differences in the pyroptosis (Fig. 12A and B) and apoptosis rates (Fig. 12A and C) between the Axl^−/−^ macrophages and control macrophages. We also verified the expression of PI3K-p110, cleaved caspase-1, and GSDMD-N after JEV infection and LY294002 treatment by immunoblotting, the results of which were consistent to those of IF staining (Fig. 12G to I, Fig. 13H and I). Taken together, these results suggest that Axl impedes the pyroptosis and apoptosis of JEV-infected peritoneal macrophages via activating PI3K-Akt signaling.

### IL-1α antagonist prevents JE pathogenesis.

**FIG 14:**
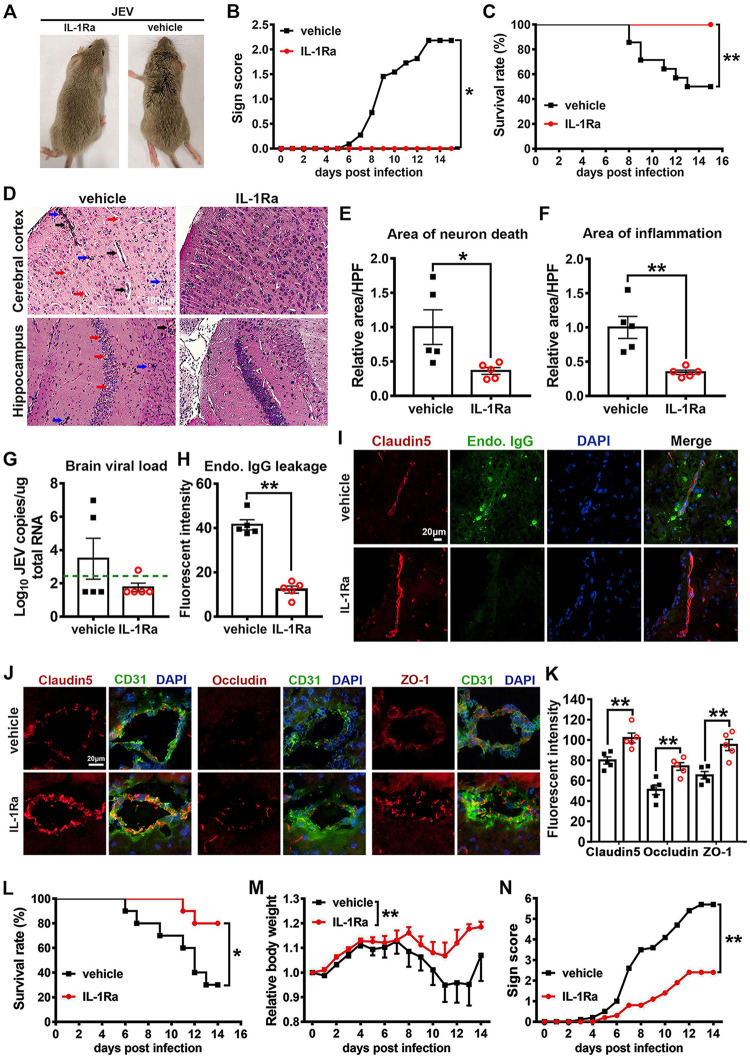
Effects of an IL-1α antagonist on JEV infection. Four-week-old Axl^−/−^ mice received an i.p. injection of recombinant rat IL-1Ra (10 μg per mouse, *n* = 13) or vehicle (sterile normal saline, *n* = 14) 1 h prior to receiving an i.p. injection of 10^4^ PFU of JEV and then were treated with IL-1Ra (5 μg per mouse) or vehicle every 2 days. (A) Representative image showing clinical manifestations in the IL-1Ra- and vehicle-treated mice with JEV infection. The IL-1Ra-treated mice showed no apparent signs of JE, while vehicle-treated mice displayed typical signs of JE, such as ruffled fur and limb paralysis. (B) Sign scores of IL-1Ra- and vehicle-treated mice with JEV infection, which were compared by rank-sum test. (C) Survival curves, which were compared by log-rank test. (D) Pathological changes in cerebral cortex and hippocampus at 7 dpi; red arrow denotes neuron death, blue arrow denotes inflammatory cell infiltration, and black arrow denotes angiectasis; scale bar = 100 μm. (E and F) Quantification of the area of neuron death (E) and the area of inflammatory cell infiltration (F) in cerebral cortex, normalized to the average of vehicle-treated mice; *n* = 5, and the data were compared by unpaired *t* test. (G) Viral load in mouse brains at 7 dpi; green dashed line denotes the limit of detection; *n* = 5, and the data were compared by Mann-Whitney test. (H) Quantification of the endogenous IgG leakage into brain parenchyma at 7 dpi; *n* = 5, and the data were compared by unpaired *t* test. (I) Representative image delineating endogenous IgG (green) leakage into brain parenchyma at 7 dpi. Claudin-5 (red) was used to position BBB; DAPI was used to show nucleus; scale bar = 20 μm. (J) Representative images depicting brain claudin-5, occludin, and ZO-1 (red); CD31 (microvascular endothelial cell marker, green); and DAPI (nucleus, blue); scale bar = 20 μm. (K) Quantification of the fluorescent intensity of claudin-5, occludin, and ZO-1 in brain; *n* = 5, and the data were compared by unpaired *t* test. (L to N) Four-week-old wild-type BALB/c mice received an i.p. injection of recombinant rat IL-1Ra (10 μg per mouse, *n* = 10) or vehicle (sterile normal saline, *n* = 10) 1 h prior to receiving an i.p. injection of 10^5^ PFU of JEV and then were treated with IL-1Ra (5 μg per mouse) or vehicle every 2 days. (L) Survival curves, which were compared by log-rank test. (M) Body weight changes, which were compared by two-way ANOVA. (N) Scores of the infection signs, which were compared by rank-sum test. All the data are expressed as the means ± SEM; *, *P* < 0.05; **, *P* < 0.01; each result is the representative of three independent experiments.

Since IL-1α promoted JE pathogenesis, we wondered if its antagonist could hinder the incidence of JE. To test this hypothesis, Axl^−/−^ mice were challenged with 10^4^ PFU of JEV and then treated with IL-1Ra (a natural IL-1α antagonist) or vehicle (sterile normal saline) every 2 days. The IL-1Ra-treated mice showed no occurrence of JE or mortality (0/13), whereas the vehicle-treated mice showed severe signs of (Fig. 14A and B) and increased mortality by JE (7/14) (Fig. 14C), suggesting the IL-1α antagonist potently reduced the incidence of JE. Next, we assessed the pathological changes in the brain, observing that JEV infection in the mouse brain caused neuron death, angiectasis, and inflammatory cell infiltration. Compared with the vehicle-treated mice, the IL-1Ra-treated mice showed significantly fewer areas of neuron death (Fig. 14D and E) and inflammatory cell infiltration (Fig. 14D and F), suggesting that the IL-1α antagonist potently relieved brain lesions during JEV infection. To elucidate the mechanism by which IL-1Ra protects mice from JE, we assessed the viral load and BBB permeability in mouse brains, observing that the IL-1Ra-treated mice exhibited reduced viral loads in the brain (Fig. 14G) and significantly decreased endogenous IgG leakage into the brain parenchyma (Fig. 14H and I). These data indicate that IL-1Ra may prevent JE pathogenesis by inhibiting increased BBB permeability and inhibiting JEV invasion of the brain during infection.

Finally, we assessed the expression and distribution of brain claudin-5, occludin, and ZO-1, observing that in the vehicle-treated mice, the expression of claudin-5 and occludin were significantly reduced and exhibited a sparse distribution along the microvascular endothelial cells (Fig. 14J). The expression of ZO-1 was also reduced, and its distribution became dispersed (Fig. 14J). Notwithstanding, in the IL-1Ra-treated mice, the expression and distribution of the three TJ proteins were comparable to that observed in the uninfected mice, where they were regularly spaced at the interfaces of microvascular endothelial cells (Fig. 14J). The above results suggest that the IL-1α antagonist can efficiently safeguard the expression and distribution of TJ proteins. In summary, the IL-1α antagonist protected mice from developing JE by reducing BBB permeability and viral invasion into brain during JEV infection.

We also verified the effect of the IL-1α antagonist on JE pathogenesis in wild-type BALB/c mice. BALB/c mice were administered IL-1Ra (*n* = 10) or vehicle (*n* = 10) in the same manner as described for the Axl^−/−^ mice and then were challenged with an even higher dose of JEV (10^5^ PFU per mouse). Compared with the vehicle-treated mice, the IL-1Ra-treated mice showed significant reductions in mortality, body weight loss, and sign scores after JEV infection (Fig. 14K to M), suggesting that the IL-1α antagonist also impedes JE pathogenesis in wild-type BALB/c mice.

## DISCUSSION

In this study, we discovered that Axl deficiency causes increased mortality in mice infected with JEV. Axl deficiency did not directly affect JEV replication; rather, it significantly increased the neuroinvasion of JEV, which was correlated with an enhanced BBB permeability. IL-1α, which was primarily produced by the JEV-infected peritoneal macrophages, was shown to be the key mediator that induced BBB breakdown and promoted JEV neuroinvasion at the early phase of infection. Furthermore, an IL-1α antagonist effectively reduced the incidence of severe JE by antagonizing JEV-induced brain TJ impairment and increased BBB permeability. The results of our study suggest that Axl is a protective factor that reduces the incidence of JE and that IL-1α promotes early JEV neuroinvasion.

Axl is a crucial and complicated host factor in flaviviral infection. Although many studies have proposed that Axl serves as an entry receptor for several flaviviruses, accumulating evidence suggests that Axl plays unique roles in a virus type-dependent manner. For example, Axl mediates the entry of DENV (13), inhibits WNV neuroinvasion without affecting viral replication in target organs (14), and also enhances ZIKV pathogenesis in an age-dependent manner by exacerbating IL-1β production and apoptosis in microglia (18). Our results show that Axl impedes JEV neuroinvasion by antagonizing viral-induced pyroptosis in peritoneal macrophages and subsequent IL-1α release. These results further suggest that Axl plays unique roles in the infection processes of different flaviviruses.

In our study, we identified IL-1α as a pivotal mediator of JEV infection and demonstrated that it functions at an early rather than late stage of JEV infection. Cytokine screening results showed that IL-1α was the most increased proinflammatory cytokine in serum from Axl-deficient mice at 1 dpi, and it potently inhibited brain TJ protein expression and distribution, resulting in increased BBB permeability. Importantly, an IL-1α antagonist effectively restored these TJ impairments and BBB disruptions and subsequently protected mice from developing severe JE. Currently, there are few reports regarding the specific role of IL-1α in modulating BBB integrity, and they primarily focus on nonviral diseases. In an *in vitro* human BBB model, IL-1α immediately increased BBB permeability within 6 h by disrupting the distribution and expression of TJ proteins, which was accompanied by cytoskeleton alteration, and its blockage by IL-1Ra could prevent such changes (27). Gloor et al. reported that IL-1α increased BBB permeability in an *in vitro* porcine BBB model by downregulating plasma membrane-associated tyrosine phosphatase activity (28). Indeed, we also observed that IL-1α immediately downregulated the expression of claudin-5, occludin, and ZO-1 in an *in vitro* mouse BBB model, which is related to actin filament and MyD88 alterations (data not shown). In addition to acting on brain microvascular endothelial cells, IL-1α also accounted for increasing pulmonary vascular permeability by downregulating the TJ protein cadherin during acute lung injury (23). IL-1β is a homolog of IL-1α and uses the same receptor as IL-1α. Pan et al. reported that DENV infection induces IL-1β secretion from macrophages in patients and mice, and IL-1β induces vascular leakage in both *in vitro* vascular endothelial monolayers and in mice, while IL-1Ra alleviates these IL-1β-mediated effects (29). In agreement with the above studies, the results of our work suggest that IL-1α is a crucial mediator that promotes the incidence of JE by disrupting BBB integrity at a very early stage of infection. Moreover, IL-1α is a promising target for JE therapy and IL-1Ra is an effective antagonist.

In our results, pyroptosis, rather than apoptosis or necroptosis, was identified as the predominant cell death mode in JEV-infected *in situ* macrophages, suggesting its pivotal role in JEV infection. Interestingly, pyroptosis is also observed in the infection of other flaviviruses. DENV infection in macrophages induces pyroptosis together with the release of IL-1β (30, 31). ZIKV infection elicits pyroptosis in the brains of fatal microcephaly cases and increases IL-1β expression (32). In addition, WNV infection in the brain also provokes the expression of pyroptosis markers (caspase-1, IL-1β, and IL-18) (33). Elucidation of the significance of viral infection-induced pyroptosis will deepen our understanding of the nature of flavivirus infections.

Intriguingly, our investigations showed that JEV-induced pyroptosis was accompanied by the release of large amounts of IL-1α rather than IL-1β. The cleavage of pro-IL-1β into mature IL-1β is a key step in pyroptosis that promotes inflammation and is also a feature that distinguishes pyroptosis from other types of cell death (34). In contrast to IL-1β, the release of IL-1α has been largely less studied in pyroptosis. Indeed, IL-1α and IL-1β share the same receptor and have similar biological functions, but they have distinct differences in the manner they are released (35). IL-1α is typically preexpressed in cells and immediately released to serve as an alarm factor by dead cells (36). In our study, IL-1α levels increased more significantly than IL-1β in Axl-deficient mice. We isolated *in situ* peritoneal macrophages from JEV-infected mice and observed that both Axl-deficient macrophages and control macrophages expressed large amounts of IL-1α in the cytosol and nucleus. However, Axl-deficient macrophages died more readily (pyroptosis and apoptosis) than control cells after JEV infection, and the dead macrophages readily released the preexpressed intracellular IL-1α and boosted serum IL-1α levels. The results of mechanistic studies indicated that Axl could activate PI3K-Akt signaling, which downregulated the expression of cleaved caspase-1 and GSDMD-N and thus inhibited the pyroptotic death of macrophages, averting large amounts of IL-1α being released by dead cells. Since IL-1α is preexpressed in macrophages and IL-1β is merely expressed upon stimulation, IL-1α is likely to act earlier and faster than IL-1β, which may explain why IL-1α is more crucial in the early phase of JEV infection. Previous studies on pyroptosis in flaviviral infection typically focused on IL-1β and not IL-1α. Therefore, whether the release of IL-1α prior to IL-1β is a universal phenomenon during pyroptosis is an interesting issue to be addressed in future studies.

In summary, in this study, we discovered that Axl is a protective factor that impedes the occurrence of JE by suppressing IL-1α production from pyroptotic macrophages and IL-1α-induced BBB breakdown. Neuroinvasive pathogens penetrate BBB in unique ways, and our results reveal a novel mechanism in which BBB penetration is driven by IL-1α. In view of the apparent protection of IL-1α blockage in JEV infection, IL-1α can thus be a potential therapeutic target to prevent severe JE. Although the above findings are interesting, there are still some issues that need to be solved. JEV is often transmitted by mosquito bites, but our experimental system used an i.p. injection, and while macrophages are one of the primary target cells for both infection routes, whether Axl and IL-1α play the same roles in JEV infection resulting from mosquito bites or a subcutaneous route remains to be elucidated.

## MATERIALS AND METHODS

### Cells and viruses.

C6/36 cells (Aedes albopictus lava cells) were grown in RPMI 1640 medium (Gibco, USA) supplemented with 10% fetal bovine serum (FBS; PAN, Germany) and maintained at 28°C. Vero cells (African green monkey kidney cells) were raised in minimum essential medium (MEM; Gibco) supplemented with 5% FBS. bEND.3 cells (murine brain microvascular endothelial cells) were maintained in Dulbecco’s modified eagle medium (DMEM; Gibco) supplemented with 10% FBS.

JEV Beijing strain-1 (JEV) (37) and *Renilla reniformis* luciferase reporter JEV (Rluc-JEV) (38) were propagated in C6/36 cells and titrated on Vero cells by plaque assay as previously reported (39).

### Mice.

Axl-deficient (Axl^−/−^) mice were kindly provided by Greg Lemke (Salk Institute for Biological Studies, La Jolla, CA). The F_0_ Axl^−/−^ mice were generated from C57BL/6J mice and were mated with F_0_ wild-type (Axl^+/+^) C57BL/6J mice to generate F_1_ heterozygous (Axl^+/−^) mice. The F_0_ Axl^−/−^ mice were then mated with F_1_ Axl^+/−^ mice to generate F_2_ Axl^−/−^ mice (experimental group) and littermate F_2_ Axl^+/−^ mice (control group). The littermate Axl^+/−^ mice are ideal controls for Axl^−/−^ mice due to them being the same age and having similar nutritional status, living status, and genetic background. The mice were bred and maintained under a specific-pathogen-free animal facility at Capital Medical University, Beijing. The genotypes were identified using PCR, and the following three primers were used: Wt, 5′-GCCGAGGTATAGTCTGTCACAG-3′; Mut, 5′-TTTGCCAAGTTCTAATTCCATC-3′; and WtMut, 5′-AGAAGGGGTTAGATGAGGAC-3′. The sizes of PCR products are 350 bp for Axl^+/+^ mice, 350 and 200 bp for Axl^+/−^ mice, and 200 bp for Axl^−/−^ mice.

Four-week-old female BALB/c mice were purchased from Vital River Laboratories (Beijing, China) and used for validating the protection of IL-1Ra during JEV infection.

### Animal infection.

Axl^+/−^ and Axl^−/−^ mice were i.p. injected with 10^4^, 10^5^, or 10^6^ PFU of JEV in 200 μl of phosphate-buffered saline (PBS) or just 200 μl of PBS in mock-treated mice. For intracerebral infection, mice were intracerebrally injected with 10^3^ PFU of JEV in 20 μl of PBS. For bioluminescent imaging, mice were i.p. injected with 5 × 10^4^ PFU of Rluc-JEV in 200 μl of PBS.

### Scoring the signs of JEV infection.

To quantitatively analyze the severity of JEV infection, signs were scored according to the following standard: well, 0; sluggish, 1; partially ruffled fur, 2; wholly ruffled fur, 3; hunched back, 4; one limb paralysis, 5; two limb paralysis, 6; more than two limb paralysis, 7; and death, 8.

### H&E staining.

Brains of JEV-infected or mock-treated mice were collected at 7 dpi. The brains were immersed in 4% paraformaldehyde (PFA; in PBS) for paraffin embedding and sectioning. Paraffin-embedded brains were sagittally sectioned into 5-μm slides, which were then stained with hematoxylin and eosin (H&E) according to a standard protocol. The analyses of H&E staining results were completed by using Image-Pro Plus 6.0 software.

### IF staining.

For immunofluorescent (IF) staining, tissue sections or cell monolayers were subjected to membrane permeability with 0.5% Triton (in PBS) for 5 min and blockage with 1% bovine serum albumin (BSA) for 1 h; then, they were incubated with specific primary antibodies (1:250 to 1:300) at 4°C overnight and incubated with Alexa Fluor 488-labeled donkey anti-mouse IgG antibody (1:500; catalog number A21202; Thermo) and/or Alexa Fluor 594-labeled donkey anti-rabbit IgG antibody (1:500; A21207; Thermo) at room temperature for 50 min. The primary antibodies used in IF staining include anti-JEV serum (self-made), anti-NeuN (ab104224; Abcam), anti-GFAP (ab7260; Abcam), anti-Iba1 (10904-1-AP; Proteintech), anti-CD31 (ab9498; Abcam), anti-claudin-5 (AF0130; Affinity Biosciences), anti-Occludin (71-1500; Thermo), anti-ZO-1 (61-7300; Thermo), anti-F4/80 (ab100790; Abcam), anti-IL-1α (DF6893; Affinity Biosciences), anti-GSDMD-N (AF4013; Affinity Biosciences), anti-RIPK1 (AF7377; Affinity Biosciences), anti-caspase-8 (ab227430; Abcam), anti-caspase-9 (ab202068; Abcam), anti-caspase-3 (9664S; Cell Signaling Technology [CST]), anti-PI3K-p110 (AF5112; Affinity Biosciences), and anti-pAkt (AF0016; Affinity Biosciences). The analyses of IF staining results were accomplished using Image-Pro Plus 6.0 software. Specifically, the IF staining images were collected under fixed excitation light intensity and exposure time. For each slide from a mouse, at least eight random scopes were collected under appropriate magnification. Then, the original images were subjected to fluorescent intensity gauge for specific staining signals by using the count/size function of Image-Pro Plus 6.0 software. The selected measurements were area and density (red). The mean density of all software-identified specific staining signals was extracted as the fluorescent intensity for an image (scope). The average fluorescent intensity of at least eight images (scopes) for each slide was defined as the fluorescent intensity for an independent mouse.

### RNA extraction in serum and major target organs.

Serum, brain, spleen, liver, and kidney were collected at 1, 4, and 7 dpi. The total RNA in serum was extracted using the EasyPure viral DNA/RNA kit (ER201-01; TransGen). The total RNA in organs was extracted using the TransZol reagent (ET101-01; TransGen).

### Quantitative real-time PCR.

For viral load detection, a previously reported (40) 6-carboxyfluorescein (FAM)-6-carboxytetramethylrhodamine (TAMRA) probe-based quantitative real-time PCR (qRT-PCR) method was used to quantify JEV genome RNA copies in the cell supernatant, serum, and brain. We also developed an SYBR-based qRT-PCR method to reflect viral loads in spleen, liver, and kidney (in these organs, viral loads were quite low), and the results were expressed as JEV copies/glyceraldehyde-3-phosphate dehydrogenase (GAPDH) copies. The primer set for JEV is sense, 5′-CGTTTCGTGCTGGCTCTTAT-3′; and antisense, 5′-CCAAGTTCTCGTTTGAAACT-3′. The primer set for GAPDH is sense, 5′-CCTGGAGAAACCTGCCAAGT-3′; and antisense, 5′-GGAGTTGCTGTTGAAGTCGC-3′. For brain tight junction RNA quantification, a previously reported (41) qRT-PCR method was modified and used. Specifically, the primer sets for brain tight junctions (claudin-5, occludin, and ZO-1) were kept the same as the reported method, while the normalization control was GAPDH in our system rather than HRPT (hypoxanthine phosphoribosyltransferase gene) used by the reported method.

### Isolation, culture, and infection of primary cells.

To isolate peritoneal macrophages, 4-week-old mice were euthanized and disinfected, followed by i.p. injection with 5 ml of DMEM, abdominal massage for 3 min, and incubation for 7 min. Then, the injected DMEM in abdominal cavity was collected and centrifuged at 2,000 rpm for 10 min. The resulting cell pellet was washed and recovered in DMEM supplemented with 10% FBS. The cells were cultured for 4 h and then washed with PBS to remove nonadherent cells; finally, fresh DMEM supplemented with 10% FBS was added. Isolated peritoneal macrophages were stained with an anti-F4/80 antibody to monitor the cell purity. And using this method, the macrophage purity can be no less than 98%. For infection, peritoneal macrophages were infected with JEV at a multiplicity of infection (MOI) of 1 for 1.5 h.

To isolate splenocytes, the spleen was collected from mice and ground on a 200-mesh filter. The filtrate was collected and subjected to density gradient centrifugation with 1× mouse lymphocyte separation medium (DKW33R0100; Dakewe). The splenocytes were cultured with 10% FBS RPMI 1640 medium. For infection, splenocytes were infected with JEV at an MOI of 1 for 1.5 h.

### BLI of Rluc-JEV infection in mice.

Axl^+/−^ (*n* = 15) and Axl^−/−^(*n* = 15) mice were i.p. injected with 5 × 10^4^ PFU of Rluc-JEV. The mice were subjected to bioluminescence detection by a bioluminescence detecting system (PerkinElmer, USA) at 2, 4, 5, 8, and 10 dpi. Each mouse was i.p. injected with 200 μl of 0.148 mM ViviRen substrate (P1232; Promega) 10 min prior to immediate imaging for 90 s at 8 bins.

### BBB permeability evaluation.

Three independent methods were used to measure BBB permeability, namely, Evans blue (EB) leakage test, sodium fluorescein (NaF) leakage test, and endogenous IgG leakage detection.

For EB brain leakage test, mice were intravenously injected with 100 μl of a 2% EB (E808783; Macklin) solution (in normal saline). After 45 min to allow for the circulation of EB, the mice were anesthetized with 200 μl of a 3.5% chloral hydrate solution. The anesthetized mice were then transcardially perfused with 30 ml of normal saline to completely remove EB in circulation. The mouse brains were then collected, weighed, and homogenized in dimethylformamide (DMFA) (200-mg brain tissues/500 μl DMFA). The homogenates were kept at 60°C for 24 h, followed by centrifugation at 12,000 × *g* for 5 min. A total of 100 μl of the resulting supernatant was transferred into a 96-well plate to measure absorbance at 620 nm. The content of EB in the brain was calculated according to the standard curve and expressed as EB content (μg) per brain tissues (g). To facilitate the linear correlation analysis between brain viral load (log_10_) and BBB permeability, the EB content was normalized to the average EB content of Axl^+/−^ mice at 0 dpi.

A NaF leakage test was performed according to a method previously reported (42). Briefly, mice were i.p. injected with 100 μl of 100 mg/ml NaF. Then, circulating blood was gleaned to separate serum. The serum (60 μl) was mixed with 60 μl of 15% trichloroacetic acid (TCA) and centrifuged. The supernatant was then mixed with 30 μl of 5 M NaOH and 7.5% TCA, and NaF content was measured as described below. Euthanized mice were transcardially perfused with 30 ml of normal saline. The brain tissues (200 mg) were homogenized in 7.5% TCA (600 μl) and centrifuged to separate supernatant. The supernatant (120 μl) was mixed with 30 μl of 5 M NaOH. NaF content in the mixture was determined using a spectrophotometer (Multiskan spectrum; Thermo Scientific) with excitation at 485 nm and emission at 530 nm. A standard curve (0 to 25 μg/ml) was used to calculate the NaF content in samples. NaF leakage into brain parenchyma was expressed as brain NaF content/serum NaF content to normalize different blood levels of the dye.

For endogenous IgG detection, brains were embedded in OCT and cryo-sectioned into 6-μm slides, anti-claudin-5 antibody was used to demonstrate BBB, and Alexa Fluor 488-labeled donkey anti-mouse IgG was used to visualize endogenous IgG.

### Multiplex immunoassays and enzyme-linked immunosorbent assay.

Axl^+/−^ and Axl^−/−^ mice were i.p. injected with 10^4^ PFU of JEV, and sera were collected at 1 dpi and 7 dpi. Supernatants from cultured cells were collected at indicated time points. The sera were subjected to a bead-based immunoassay (Aimplex multiplex immunoassays for flow; Beijing Quantobio Co., Ltd.) to measure a panel of cytokines (IL-1α, IL-1β, IL-2, IL-4, IL-6, IL-10, TNF-α, IFN-γ, CCL2, and CCL5). Specially, the contents of IL-1α in peritoneal washes, supernatants of peritoneal macrophages, and other cells were gauged by enzyme-linked immunosorbent assay (ELISA) (ELM-IL1a; Raybiotech).

### R428 treatment.

bEND.3 cells were pretreated with culture medium containing 1 μM R428 (21523; Cayman Chemical) or vehicle (0.1% DMSO) for 30 min and then infected with JEV at an MOI of 1 for 1.5 h in the presence of 1 μM R428 or vehicle. After infection, the viral inoculates were completely removed and replaced with fresh culture medium containing 1 μM R428 or vehicle. The cell supernatants were collected at 24 hpi and subjected to IL-1α measurement.

### Trypan blue staining.

The isolated peritoneal macrophages were stained with 0.04% of a TB solution (final concentration) for 3 min and then immediately smeared on a slide for cell death counting and photographing.

### TUNEL staining.

*In situ* peritoneal macrophages were isolated from JEV-infected mice and immediately made into smears, followed by TUNEL staining with the one-step TUNEL apoptosis assay kit (C1086; Beyotime).

### Immunoblotting.

The isolated peritoneal macrophages were lysed with 100 μl of 1× cell lysis buffer (9803S; CST), ultrasonicated, mixed with 25 μl of 5× SDS loading buffer (P0015; Beyotime), and finally boiled for 15 min. The resulting samples were subjected to an immunoblotting protocol (43) for the detection of specific proteins.

### Drug administration.

Mice (Axl^−/−^) in the IL-1α- or IL-6-treated group were i.p. injected with 200 ng of a recombinant mouse IL-1α (50114-MNAE, Sino Biological) or 60 ng of a recombinant mouse IL-6 (50136-MNAE; Sino Biological) per mouse 30 min prior to i.p. injection with 10^4^ PFU of JEV; then, a daily i.p. injection of 200 ng of IL-1α or 60 ng of IL-6 was administered. Mice (including wild-type BALB/c mice and Axl^−/−^ mice) in IL-1Ra-treated group were i.p. injected with 10 μg of a recombinant rat IL-1Ra (a natural IL-1α antagonist; 80073-R01H; Sino Biological) per mouse 1 h prior to i.p. injection with 10^4^ PFU of JEV; then, an every-2-day i.p. injection of 5 μg of IL-1Ra was administered. Mice (including wild-type BALB/c mice and Axl^−/−^ mice) in vehicle-treated group were i.p. injected with 200 μl of sterile normal saline 30 min prior to i.p. injection with 10^4^ PFU of JEV; then, a daily i.p. injection of 200 μl of sterile normal saline was administered. Axl^+/−^ mice were i.p. injected with 1.25 mg of LY294002 (PI3K inhibitor; HY-10108; MCE) per mouse 1 h prior to i.p. injection with 10^4^ PFU of JEV. At 24 hpi, peritoneal macrophages were isolated and made into smears for cell death studies.

### Statistical analysis.

The quantitative data were expressed as mean ± SEM, and all the statistical analyses were performed on GraphPad Prism 7.00 software. Student’s *t* test or Welch’s correction and Mann-Whitney test were used to compare quantitative data between two groups. Two-way analysis of variance (ANOVA) and multiple *t* tests were used to compare grouped quantitative data. Specially, the log-rank test was used to compare survival curves. Fisher’s exact test was used to compare the probability of JEV entry into the brain. The rank-sum test was used to compare sign scores. A linear regression model was used to analyze the correlation between brain viral load and BBB permeability and the correlation between IL-1α content in serum and IL-1α content in peritoneal wash. Comparisons were considered statistically different when the *P* value was <0.05.

### Ethical statement.

All the animal experiments were reviewed and approved by the Experimental Animal Welfare and Animal Ethics Committee of Capital Medical University, Beijing, China (permission code, EEI-2015-048; permission date, 20 April 2015).
